# AGHE as an Incubator for Interdisciplinary Education

**DOI:** 10.1093/geroni/igaa057.1770

**Published:** 2020-12-16

**Authors:** Thomas Teasdale, Judith Howe, Carol Rogers

**Affiliations:** 1 University of Oklahoma Health Sciences Hudson College of Public Health, Oklahoma City, Oklahoma, United States; 2 Icahn School of Medicine at Mount Sinai, Bronx, New York, United States; 3 University of Oklahoma Health Sciences Center, Reynolds Center for Geriatric Nursing Excellence, Oklahoma City, Oklahoma, United States

## Abstract

For several decades, the history of interdisciplinary education and the development of AGHE initiatives have been closely linked. The need to educate colleagues on methods and benefits of interdisciplinary/ interprofessional cooperation toward service and research of aging has never waned. In this presentation we (a) highlight how AGHE has performed as a potent incubator for progress in this area and (b) use a few examples to illustrate how notable resulting efforts have improved geriatric care. For example, early and significant infusion of federal funds for gerontology training programs supported multi-disciplinary university-based centers, the Veterans Health Administration created interprofessional geriatric training programs, foundations such as John A. Hartford and Josiah Macy founded team training and interprofessional education programs, and the Health Resources and Services Administration funded Geriatric Education Centers and Geriatric Workforce Enhancement Programs. Efforts to advance interdisciplinary/interprofessional education have been fruitful and AGHE’s role as an incubator continues to evolve.

